# Fabrication of high-Q lithium niobate microresonators using femtosecond laser micromachining

**DOI:** 10.1038/srep08072

**Published:** 2015-01-28

**Authors:** Jintian Lin, Yingxin Xu, Zhiwei Fang, Min Wang, Jiangxin Song, Nengwen Wang, Lingling Qiao, Wei Fang, Ya Cheng

**Affiliations:** 1 State Key Laboratory of High Field Laser Physics, Shanghai Institute of Optics and Fine Mechanics, Chinese Academy of Sciences, Shanghai 201800, China; 2 State Key Laboratory of Modern Optical Instrumentation, Department of Optical Engineering, Zhejiang University, Hangzhou 310027, China; 3 University of Chinese Academy of Sciences, Beijing 100049, China; 4 School of Physical Science and Technology, ShanghaiTech University, Shanghai 200031, China

## Abstract

We report on fabrication of high-Q lithium niobate (LN) whispering-gallery-mode (WGM) microresonators suspended on silica pedestals by femtosecond laser direct writing followed by focused ion beam (FIB) milling. The micrometer-scale (diameter ~82 μm) LN resonator possesses a Q factor of ~2.5 × 10^5^ around 1550 nm wavelength. The combination of femtosecond laser direct writing with FIB enables high-efficiency, high-precision nanofabrication of high-Q crystalline microresonators.

In whispering-gallery-mode (WGM) microresonators, the total internal reflection along the smooth circular peripheries can lead to high quality (Q) factors and small volumes (V) for inducing dramatic enhancement of light fields. Thanks to the excellent properties, WGM microresonators have been frequently employed in a broad range of applications such as microlasers^1^, sensing^2^, optomechanics^3^, nonlinear optics^4^,^5^, and cavity quantum electrodynamics^6^. At present, a variety of semiconductor and silica WGM microresonators have been fabricated based on semiconductor lithography approach, in which either second (χ^(2)^) or third order (χ^(3)^) nonlinear optical process have been demonstrated with pronounced conversion efficiency^7^,^8^,^9^,^10^. However, from the application point of view, the semiconductor microresonators frequently suffer from relative high absorption loss due to their small bandgaps, whereas the silica microresonators have a vanishing χ^(2)^ and a low χ^(3)^. For overcoming these difficulties, development of microresonators based on advanced nonlinear optical materials is highly desirable.

Dielectric crystalline WGM resonators have shown great promise as the next generation nonlinear sources of both classical and nonclassical light, owing to their unique characteristics including high nonlinear optical coefficients, low intrinsic absorption loss, and large transparent windows^11^,^12^,^13^. In particular, as an important ferroelectric nonlinear crystalline material, lithium niobate (LN) crystal has received significant attention because of its high second order nonlinear and electro-optic coefficients^14^. However, owing to the technical difficulties related to the material growth and the lithographic fabrication on LN substrate, high-Q LN resonators are now typically realized using mechanical polishing whose sizes are limited to millimeter-scale^15^. Realization of high-Q on-chip sub-millimeter LN resonators remains a challenge^14^,^16^.

We establish a new technique to fabricate on-chip high-Q sub-millimeter LN microresonators. The concept of our technique is briefly introduced in Fig. 1. We fabricate the microresonator on an LN thin film which is formed by bonding an ion-sliced LN thin film onto a LN substrate with a sandwiched silica layer^17^. As schematically illustrated in Fig. 1, the procedure of fabrication consists of (1) femtosecond laser ablation of the thin film sample which is immersed in water to form a cylindrical post with a total height of ~15 μm^18^,^19^, (2) smoothing the peripheries of the cylindrical post by FIB milling^20^, (3) chemical wet etching of the fabricated sample in a solution of 5% hydrofluoric (HF) diluted with water to form the freestanding LN microdisk on silica pedestal by selectively removing the silica layer under the LN thin film, and (4) high temperature annealing of the sample to further promote the Q-factor.

Using a fiber taper coupling measurement^21^, we determine that the Q factor of a LN microdisk with a diameter of 82 μm can reach ~2.5 × 10^5^. It should be noticed that for sub-100 μm-diameter LN microresonators, it is very difficult to achieve a Q-factor higher than 10^5^, to the best of our knowledge^14^,^16^. Our technique opens the new route for fabricating novel crystalline microresonators for on-chip photonics applications.

## Results and Discussion

### Characterization LN microresonator

**Figure 2:**
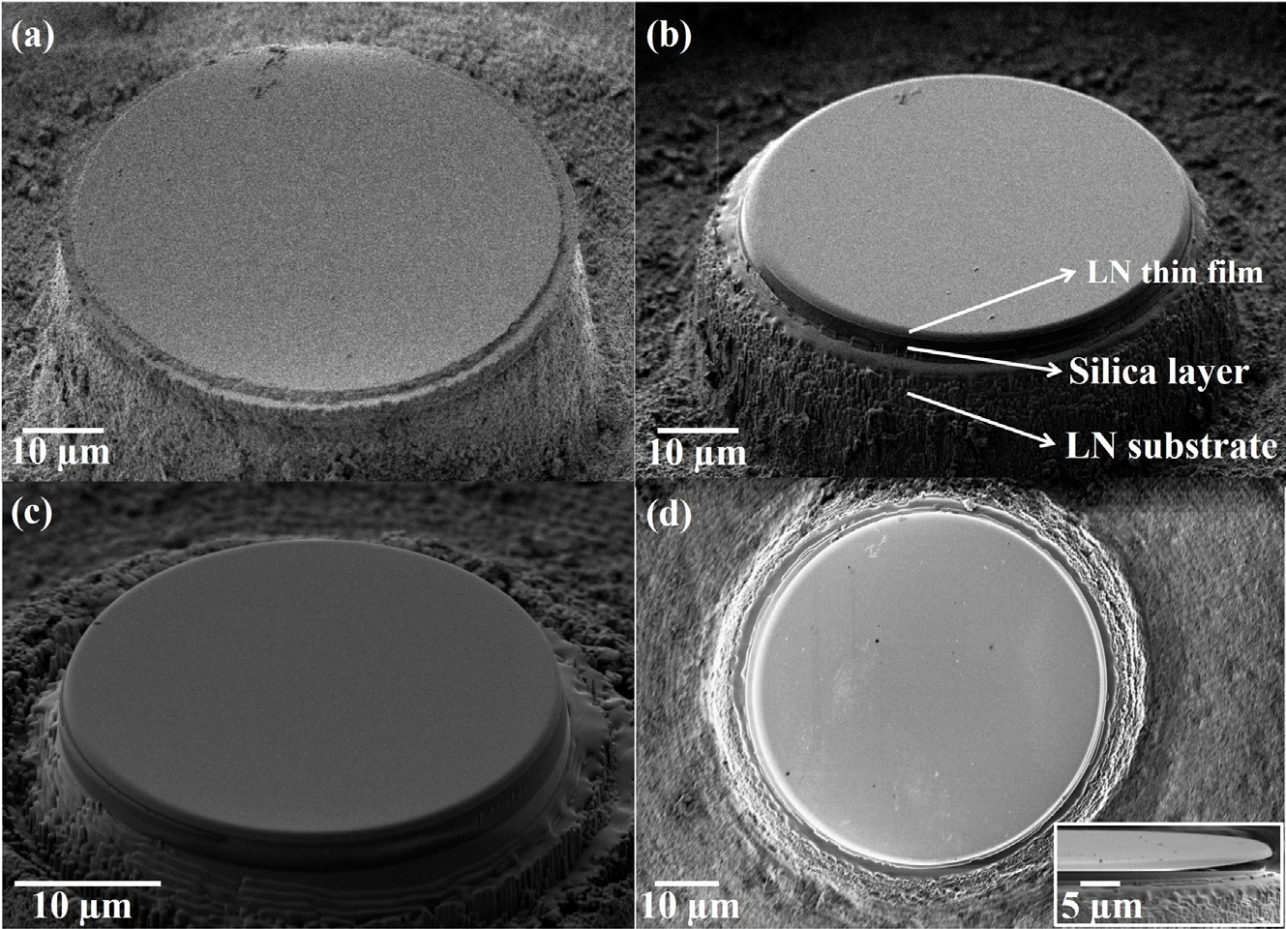
(a) SEM image of a cylindrical post formed after femtosecond laser ablation; (b) and (c) SEM images of two cylindrical posts with diameters of 55 μm and 33 μm, respectively, after the FIB milling; (d) SEM image (top view) of the 55-μm diameter microresonator after the chemical etching and annealing. Inset in (d): side view of the microresonator, showing the freestanding edge of the LN microresonator.

The details on the fabrication of LN microresonator can be found in **Methods**. The thicknesses of the LN thin film and silica layer were 700 nm and 2 μm, respectively. Briefly speaking, we first fabricated a cylindrical post with a diameter of ~59 μm and a total height of ~15 μm (corresponding to a cutting depth of 12 μm into the LN substrate beneath the silica), which is shown in Fig. 2(a), by femtosecond laser micromachining. Since the roughness on the laser ablated sidewall of the cylindrical post is on the order of a few tens of nanometers, which is too poor for high-Q microresonator application, FIB milling was used to smooth the periphery of the cylindrical post. The FIB milling was operated twice, beginning with a coarse milling and followed with a fine one. In the coarse milling, a 30-keV ion beam with a beam current of 4 nA was used to polish the periphery; whereas in the fine milling, the beam current was reduced to 1 nA. The milling was stopped at a depth of 3 μm from the top surface. The total FIB milling process took ~15 min. After the FIB milling, the diameter of the microresonator was reduced to 55 μm. Most importantly, the LN microresonator shows a highly smooth edge, as evidenced in its scanning electron microscope (SEM) image (Fig. 2(b)). Figure 2(c) shows another cylindrical post with a smaller diameter of 33 μm after the FIB milling. In principle, microresonators of diameters below 10 μm can be fabricated using our technique. It should be noted that the FIB process frequently induces the creation of lattice defects (i.e., vacancies and atomic nuclei), leading to the formation of amorphous material due to keV ion beam side dose or lateral ion straggle at the periphery of the microresonator. However, such defects are not critical in our experiments, since no free carrier is involved in the nonlinear generation process.

To form the freestanding microresonator (i.e., a thin disk sitting on top of a micro-pedestal), the silica layer sandwiched between the LN thin film and the LN substrate needs to be selectively etched to form the micro-pedestal under the LN microdisk. Therefore, the sample was immersed in a solution of 5% HF diluted with water for 8 min to form the silica pedestal. The upper LN microdisk suspended on the pedestal serves as the high-Q microresonator.

At last, a thermal annealing (500°C for 2 hrs in air) was applied to further smooth the rim of the LN thin disk. It should be mentioned that since the top and bottom surfaces of the LN thin film naturally possess an ultra-high smoothness with a surface roughness as low as 0.35 nm^17^, a high Q factor of the LN microresonator can be ensured after smoothing its rim. As shown by the side view of the freestanding microresonator in the inset in Fig. 2(d), an air gap between the LN microdisk and LN substrate can clearly be seen.

**Figure 3:**
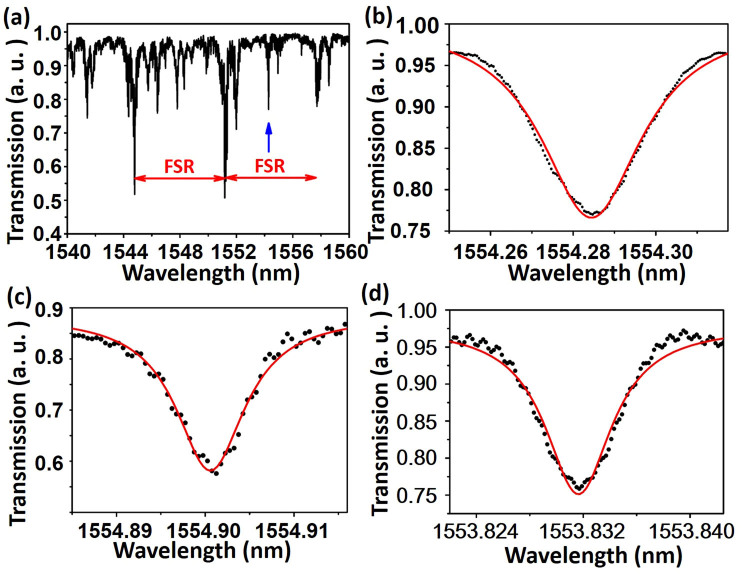
(a) Transmission spectrum of the fiber taper coupled with the microresonator (diameter ~55 μm) before annealing, (b) Lorentzian fit (red solid line) of measured spectrum around the resonant wavelength (indicated by the left blue arrow inFig. 3(a)) at 1554.28 nm (black dotted line), showing a Q factor of 5.2 × 10^4^, (c) Lorentzian fit (red solid line) of measured transmission spectrum of the microresonator (diameter ~55 μm) after annealing around the resonant wavelength at 1554.90 nm (black dotted line), showing an improved Q factor of 1.6 × 10^5^, (d) Lorentzian fit (red solid line) of measured transmission spectrum of the microresonator (diameter ~82 μm) after annealing around the resonant wavelength at 1553.83 nm (black dotted line), showing an improved Q factor of 2.5 × 10^5^.

To measure the Q-factor of the fabricated LN microresonators, an evanescent fiber taper coupling method was employed (for details, see **Methods**). The transmitted spectrum measured from the output end of the fiber taper showed a series of sharp dips at the WGM resonant wavelengths. In these measurements, coarse scans over a wide wavelength span were first performed to decide the WGM resonant wavelengths. As an example, the transmission spectrum obtained with the coarse scan with a step size of 1 nm for the 55 μm LN microresonator without annealing is shown in Fig. 3(a). After the coarse scan, fine scans with a step size of 0.5 pm around the resonant wavelengths were performed to measure the linewidths of the dips indicated by the Lorentzian fit.

The transmission spectra of the fiber taper coupled to the 55 μm microresonators before and after the annealing are shown in Figs. 3 (b)–(c). The resonance at 1554.28 nm wavelength showed a loaded Q factor of 5.2 × 10^4^ (Fig. 3(b)) of the microresonator before the annealing. After the annealing, the Q factor of the same microresonator was significantly improved to 1.6 × 10^5^ around the resonant wavelength of 1554.90 nm, as evidenced in Fig. 3(c). Furthermore, the Q factor of the microresonator with a diameter of 82 μm was measured to be 2.5 × 10^5^ after the annealing process, as indicated in Fig. 3(d). The result shows that the Q factor increases with the increasing diameter of the fabricated LN microresonator, as a result of radiation loss at the curved surface which has been well described by A. M. Armani et al^22^.

In conclusion, we demonstrate the fabrication of high-Q on-chip LN microresonators on single crystal LN thin film wafer by femtosecond laser 3D micromachining. The Q factor is measured to be 2.5 × 10^5^ around 1550 nm wavelength. Since our technique uses high precision ablation of materials with femtosecond laser pulses, it is intrinsically material insensitive. In fact, in the past few years, we have fabricated high-Q optical microresonators in various materials such as fused silica^23^, an active Nd: glass^19^, and CaF_2_ crystal^20^. It should be noticed that although femtosecond laser micromachining allows for efficient and precision ablation of dielectric materials, the surface roughness is typically too high for high-Q microresonator applications. The incorporation of FIB milling provides an ideal solution without spoiling the flexibility in femtosecond laser microfabrication. We envisage that our technique can be extended for fabricating high-Q on-chip microresonators on various types of dielectric materials.

## Methods

### Fabrication of LN microresonator

A Ti: sapphire femtosecond laser source (Coherent, Inc., center wavelength: ~800 nm, pulse width: ~40 fs, repetition rate: 1 kHz) was used for fabricating LN microresonators. A variable neutral density filter was used to carefully adjust the average power. An objective lens with a numerical aperture (NA) of 0.80 was used to produce the tightly focused spot with a diameter of ~1 μm. The laser beam then focused into the LN thin film sample immersed in water. The sample was mounted on a computer-controlled XYZ translation stage with 1-μm resolution. A charged coupled device (CCD) connecting with the computer was installed above the objective lens to monitor the fabrication process in real time.

A layer-by-layer annular ablation from the top surface to internal substrate with 1 μm interval between the adjacent layers was adopted, so that the ablation always occurred at the interface between the water and the material. In this manner, the ablation debris can be more efficiently removed with the assistance of water. The laser power was chosen to be 0.35 mW for ablation in both the LN thin film and the LN substrate beneath the silica layer, whereas the laser power was raised to 1 mW for ablation in silica layer, because the ablation thresholds of LN crystal and silica glass are different.

### Measurement of Q-factor

The Q factors were measured by the fiber taper coupling method. An external cavity continuous wave tunable laser diode (New Focus, Model: 6528-LN; output power: 0.08 mW; wavelength range: 1510 nm ~ 1620 nm; step resolution: 0.1 pm) was coupled into the fiber taper. The fiber taper with a diameter of ~1 μm was home made by heating and stretching a section of a commercial optical fiber (Corning, SMF-28). A transient optical power detector was used to measure the transmission spectra at the output end of the fiber taper. The detector can record the light signal over a 100 nm-wavelength-span with 0.5 pm wavelength resolution and 0.015 dB power accuracy in less than 1 second. A piezo-stage was used to control the relative position between the microresonator and the fiber taper waist. The critical coupling between the WGMs and the evanescent field of the fiber taper can be achieved by carefully adjusting the gap between the fiber taper and the microresonator. We used two CCD cameras whose optical axes are arranged to be perpendicular and parallel to the equatorial plane of the microresonators to simultaneously acquire both the side-view and top-view images of the microresonators coupled with the fiber taper. In the fine scan for measuring the Q factor, a slow scan speed of 2 nm/s was chosen.

## Author Contributions

J.L., W.F. and Y.C. planned and designed the experiments. J.L., Z.F. and M.W. established the experimental setup. J.L., J.S. and N.W. fabricated the microresonator using femtosecond laser direct writing. J.L., L.Q. and Y.X. performed FIB milling. J.L., W.F. and Y.C. analyzed the data. All authors participated in the discussion of the results and the writing of the manuscript.

## Figures and Tables

**Figure 1:**
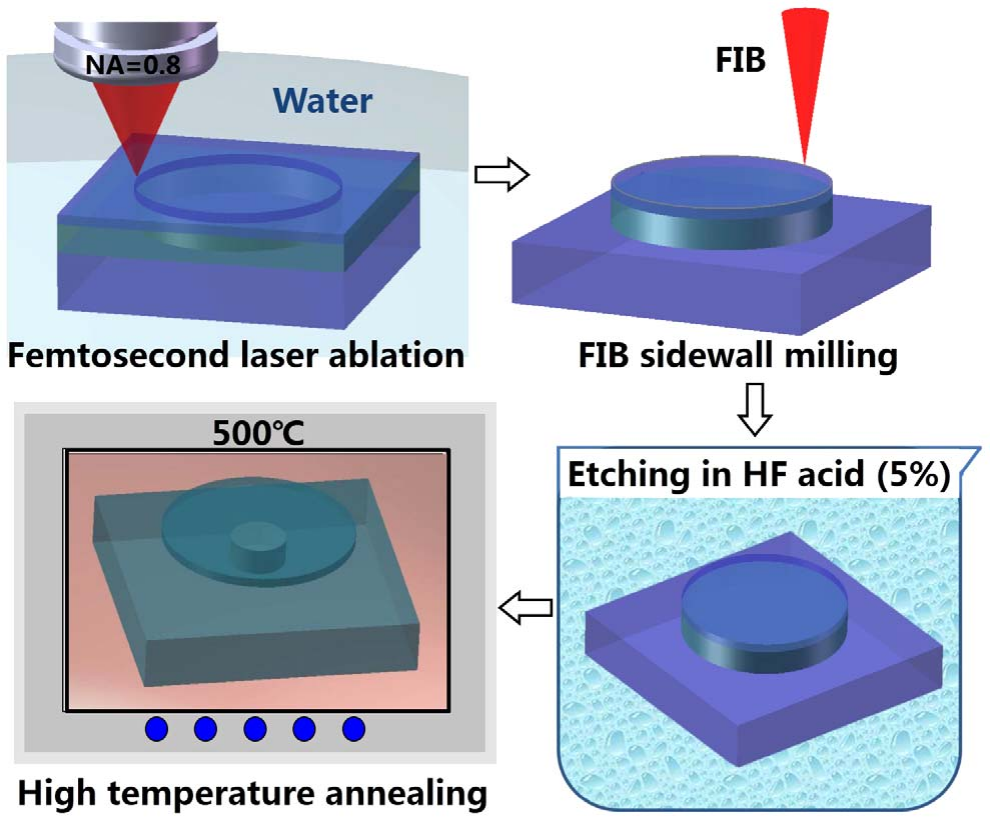
Procedures of fabrication of a LN microresonator by water-assisted femtosecond laser ablation, followed by FIB milling, selective chemical etching, and annealing.
